# Relationships Between Dry-Land Load—Velocity Parameters and In-Water Bioenergetic Performance in Competitive Swimmers

**DOI:** 10.3390/sports14010011

**Published:** 2026-01-03

**Authors:** Sofiene Amara, Anissa Bouassida, Roland van den Tillaar

**Affiliations:** 1 Research Unit, Sportive Performance and Physical Rehabilitation, High Institute of Sports and Physical Education of Kef, University of Jendouba, Kef 7100, Tunisia; anissa.bouassida@gmail.com; 2 Department of Sports Sciences and Physical Education, Nord University, 7600 Levanger, Norway

**Keywords:** critical velocity, anaerobic capacity, neuromuscular performance, bench press, swimming performance

## Abstract

Background: Neuromuscular determinants such as maximal force, maximal velocity, and upper-body power are recognized as key contributors to competitive swimming performance. However, despite the relevance of these dry-land qualities, their relationships with the physiological mechanisms underpinning in-water performance, particularly aerobic and anaerobic capacities, remain insufficiently established. Purpose: This study aimed to investigate the relationships between upper-body load–velocity profile parameters (theoretical maximal force: F_0_; theoretical maximal velocity: V_0_; and maximal power: P_max_), aerobic capacity expressed through critical velocity, and anaerobic capacity in trained swimmers. Methods: Thirty competitive male swimmers (age = 16.50 ± 0.31 years) completed an upper-body load–velocity profile test using the bench press exercise to determine F_0_, V_0_, and P_max_. Swimming performances in the 100, 200, and 400 m freestyle events were used to calculate critical velocity and anaerobic capacity based on a linear distance–time model. Pearson correlation coefficients and linear regression analyses were conducted to examine the relationships between variables. Results: P_max_ (r = 0.493, *p* = 0.006) and V_0_ (r = 0.697, *p* < 0.001) showed moderate to strong correlations with critical velocity, whereas F_0_ showed no significant association (r = 0.152, *p* = 0.422). Conversely, anaerobic capacity was strongly correlated with F_0_ (r = 0.842, *p* < 0.001) but not with V_0_ (*p* = 0.119). Regression models indicated that F_0_ explained 71% of the variance in anaerobic capacity, while V_0_ explained 48% of the variance in critical velocity. Conclusion: The findings demonstrated distinct contributions of neuromuscular qualities: speed and power-oriented parameters are associated with critical velocity, whereas maximal strength strongly associated with anaerobic capacity. Monitoring the upper-body load–velocity profile appears to be a relevant tool for individualizing dry-land training according to the aerobic and anaerobic demands of swimmers.

## 1. Introduction

Competitive swimming performance results from a dynamic interaction between the athlete’s physiological, muscular, technical, and biomechanical capabilities [1]. Over the past decade, several studies have shown that the determinants of swimming velocity include not only aerobic and anaerobic capacities but also neuromuscular characteristics such as maximal strength, power, and the load–velocity profile [2,3]. For example, it has been reported that 50 to 70% of sprint speed variability can be explained by differences in upper-body force and power production [4]. Therefore, understanding muscular factors influencing movement efficiency in swimming remains essential for optimizing performance.

Dryland training is widely used to improve strength and power in swimmers [5,6]. Recent findings indicate that explosive power-oriented programs can improve sprint performance by 3 to 8% in trained swimmers [7,8]. Among these approaches, estimating theoretical maximal force (F_0_) and theoretical maximal velocity (V_0_) derived from the linear load–velocity relationship enables classification of athletes according to their neuromuscular profile (force-dominant vs. velocity dominant) and allows individualized upper-body training [9,10]. Additionally, exercises such as the bench press, frequently used in elite swimming preparation, are considered reliable indicators of trunk and upper-limb power [11]. However, despite increasing interest in strength, velocity, and power in relation to swimming performance, their associations with the physiological mechanisms underlying in-water performance, particularly aerobic and anaerobic capacities, remain insufficiently explored.

In physiological testing for swimmers, two reference variables are commonly used: (i) critical velocity, recognized as a robust marker of aerobic capacity and endurance, and (ii) anaerobic work capacity available above critical velocity [12,13]. These metrics allow modeling of exercise tolerance, training load prescription, and performance prediction across race distances [12]. Previous research has shown moderate to strong correlations between critical velocity and performance in distances ≥200 m (r = 0.60 to 0.85) [14,15], while anaerobic capacity is also positively associated with sprint performance (r = 0.55 to 0.78) [16,17]. Despite their strategic relevance, few studies have examined how neuromuscular dryland qualities, specifically upper-body strength, maximal velocity, and power, relate to critical velocity and anaerobic capacity [18]. Addressing this gap is essential, as establishing these relationships could provide insight into the mechanistic links between strength, power, and physiological swimming performance, thereby offering a scientific basis for tailoring dryland training according to the aerobic and anaerobic requirements of swimmers.

Most existing research has focused either on the isolated effects of strength training or solely on the relationships between critical velocity, anaerobic capacity and performance. Therefore, there remains a lack of evidence regarding whether theoretical F_0_, V_0_, or higher maximal power associates differently to the aerobic (critical velocity) and anaerobic components of swimming performance. Accordingly, the purpose of this study was to examine the relationship between the load–velocity profile, bench press-derived muscular power, and two key physiological determinants of performance: critical velocity and anaerobic capacity in competitive swimmers. We hypothesized that swimmers with higher maximal velocity and power exhibit greater critical velocity values. Additionally, maximal strength and power were expected to show stronger associations with anaerobic capacity compared with critical velocity, reflecting their roles in anaerobic performance.

## 2. Materials and Methods

### 2.1. Study Design

This cross-sectional observational study was conducted to examine the relationships between upper-body load–velocity profiling and key physiological determinants of swimming performance, namely critical velocity and anaerobic capacity. All assessments were performed across two separate testing sessions spaced 48 h apart to minimize fatigue effects: the first session consisted of upper-body load–velocity testing using the bench press exercise, and the second session included pool tests to determine critical velocity and anaerobic capacity. The study protocol was approved by the Institutional Ethics Committee of the Higher Institute of Sport and Physical Education of Kef, University of Jendouba, Tunisia (protocol code: ISSEPK-0022/25), and was conducted in accordance with the ethical principles outlined in the Declaration of Helsinki.

### 2.2. Participants

Thirty trained male swimmers voluntarily participated in the study (age: 16.5 ± 0.31 years; body mass: 69.4 ± 4.9 kg; height: 176.2 ± 5.6 cm). All swimmers were classified as regional to national-level athletes, according to Tunisian and international performance standards for the 15–17-year-old category. Their performance tier was justified by their official best times recorded during the competitive season, their engagement in structured training programs comprising at least five sessions per week, and their regular participation in sanctioned regional and national competitions. Inclusion criteria were: (i) absence of acute or chronic injury affecting performance, (ii) uninterrupted participation in swimming training during the previous six months, and (iii) the ability to perform the bench press exercise. Exclusion criteria consisted of any musculoskeletal injury, respiratory or metabolic disorder, or any training interruption occurring within the previous two months. All participants received a detailed explanation of the study procedures and provided written informed consent.

### 2.3. Load–Velocity Profile and Maximal Power Assessment Using the Bench Press

The dryland testing session took place in a climate-controlled strength training facility where ambient temperature was maintained at 22.3 °C and relative humidity at 45%, in accordance with recommended environmental standards for performance testing [19].

The load–velocity profile was assessed using a Smith machine bench press to constrain bar movement to a vertical trajectory [9,10]. Following a standardized warm-up (10 repetitions at 40% 1RM, 5 repetitions at 60% 1RM, and 3 repetitions at 70% 1RM), participants performed 4–5 sets of one maximal-intent repetition with progressively increased loads ranging from 40 to 80% 1RM. Load increments were individualized to preserve the validity of the load–velocity relationship: +10 kg when mean concentric velocity remained above 0.80 m·s^−1^, and +5 kg when velocity fell below this threshold. Consequently, participants did not lift identical absolute loads but rather loads expressed as equivalent percentages of their individual estimated 1RM.

The mean bar velocity at 80% 1RM was 0.42 ± 0.05 m·s^−1^. Bar velocity was measured using a linear position transducer (GymAware, Braddon, Australia) PowerTool, Kinetic Performance, Australia). The variables F_0_, V_0_, and P_max_ were calculated using a linear regression model based on the load–velocity relationship, following recommended procedures [9,10]. A strict rest interval of 3 min was maintained between attempts. This protocol is widely used to assess neuromuscular capacities in swimmers [11,20].

### 2.4. Measurement of Critical Velocity (CV) and Anaerobic Capacity (W)

**Figure 1:**
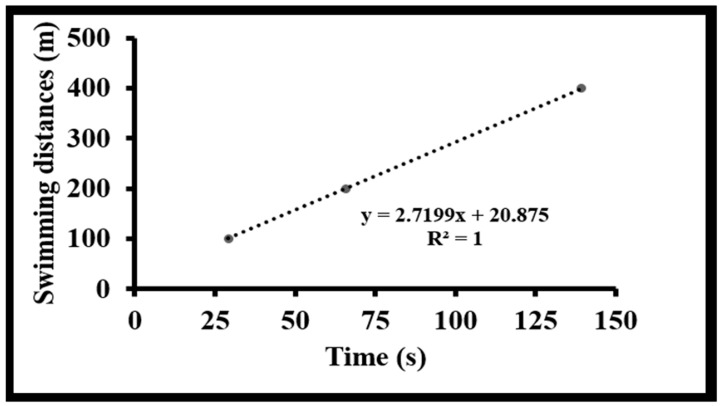
Linear regression of swimming distance versus time for swimmer 1.

Swimming performance assessment was conducted in a 25 m indoor pool under controlled environmental conditions (water temperature: 27.1 ± 0.5 °C; air temperature: 25.9 ± 0.3 °C; relative humidity: 64 ± 5%), compliant with competitive aquatic environment standards [21]. After a standardized progressive 800 m warm-up, swimmers performed three maximal timed trials in crawling over 100, 200, and 400 m in randomized order, with a minimum of 20 min of passive recovery between trials. Prior to each new trial, a brief 3 min reactivation routine consisting of light kicking and submaximal technical drills was completed to re-establish optimal readiness. Performance times were recorded by an experienced evaluator using a handheld stopwatch (SEIKO S120-4030, Tokyo, Japan). critical velocity and anaerobic capacity were determined using the linear distance–time model:

Distance = anaerobic capacity + (critical velocity × time)

where critical velocity corresponds to the slope (m·s^−1^) and anaerobic capacity to the intercept (m), as recommended by Zacca et al. [16]. This model is recognized as a standard method for assessing aerobic and anaerobic capacities in competitive swimming [15,16,17]. For illustration, swimmer 1 completed the timed trials in 29.09 s (100 m), 65.86 s (200 m), and 139.39 s (400 m). Based on the linear relationship between distance and swimming time (distance–time model: y = 2.7199x + 20.857), the derived physiological indicators resulted in a critical velocity of 2.72 m·s^−1^ and an anaerobic work capacity of 20.86 m (Figure 1).

### 2.5. Statistical Analysis

Statistical analyses were performed using SPSS software (version 26.0, IBM Corp., Armonk, NY, USA). The normality of data distribution was verified using the Shapiro–Wilk test. Descriptive statistics, including means and standard deviations, were calculated for all variables. Pearson correlation coefficients were used to examine the relationships between F_0_, V_0_, P_max_, and both critical velocity and anaerobic capacity. Correlation coefficients were interpreted using standard thresholds: r = ±0.10–0.39 (weak), ±0.40–0.69 (moderate), ±0.70–0.89 (strong), and ±0.90–1.00 (very strong). Multiple linear regression analyses were conducted to identify predictive variables of physiological performance outcomes. The level of statistical significance was set at *p* < 0.05.

## 3. Results

These descriptive characteristics of the neuromuscular and physiological variables are presented as mean ± standard deviation in Table 1. Based on the linear distance–time model, the calculated physiological determinants were critical velocity = 2.70 ± 0.13 m·s^−1^ and anaerobic capacity = 21.20 ± 1.17 m (Table 1).

**Figure 2:**
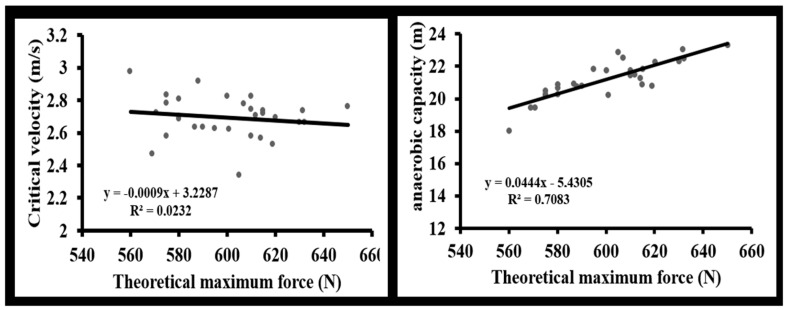
Linear regressions of theoretical maximal force (F_0_) with critical velocity and anaerobic capacity.

Correlation analyses revealed distinct associations between the force–velocity profile variables and the physiological performance determinants. Regarding critical velocity, a strong positive correlation was observed with V_0_ (r = 0.697, *p* < 0.001), while P_max_ showed a significant moderate correlation (r = 0.493, *p* = 0.006). In contrast, no significant relationship was found between F_0_ and critical velocity (r = 0.152, *p* = 0.422) (Table 2, Figure 2).

**Figure 3:**
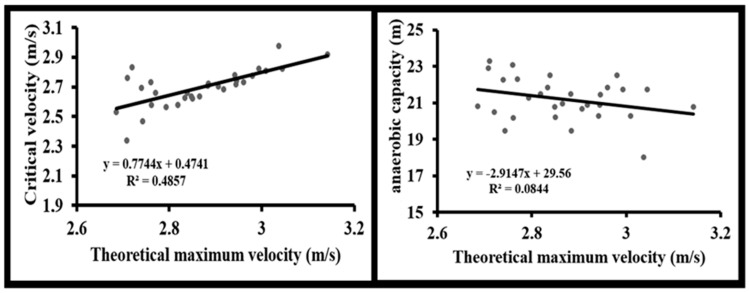
Linear regressions of theoretical maximal velocity (V_0_) with critical velocity and anaerobic capacity.

**Figure 4:**
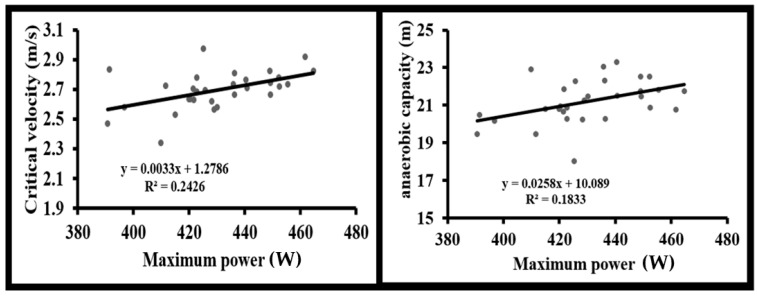
Linear regressions of theoretical maximal power (Pmax) with critical velocity and anaerobic capacity.

Conversely, anaerobic capacity was strongly associated with F_0_ (r = 0.842, *p* < 0.001). A significant moderate correlation was also found between anaerobic capacity and P_max_ (r = 0.428, *p* = 0.018), while V_0_ showed no significant association with anaerobic capacity (*p* = 0.119) (Figure 3 and Figure 4).

## 4. Discussion

The aim of this study was to examine the relationship between upper-body load–velocity profile characteristics (F_0_, V_0_, and P_max_), critical velocity, and anaerobic capacity in competitive swimmers as there was a lack of evidence regarding whether theoretical F_0_, V_0_, or higher maximal power associated differently to the aerobic (critical velocity) and anaerobic components of swimming performance. The main findings indicate that P_max_ and V_0_ show moderate to strong correlations with critical velocity, while F_0_ demonstrates no significant association. Conversely, anaerobic capacity was strongly correlated with F_0_, whereas V_0_ did not account for a significant proportion of variance in anaerobic capacity within the regression model. These results confirm the hypothesis of a differentiated relationship of the force- and velocity-oriented neuromuscular components to the physiological determinants of swimming performance.

The positive association between V_0_ and critical velocity suggests that the ability to produce high contraction velocities may contribute to improved propulsive efficiency and reduced energetic cost at a given speed. This aligns with findings indicating that performance over distances ≥200 m depends not only on aerobic capacity but also on mechanical efficiency and stroke frequency optimization mediated by neuromuscular speed [12,13,14,15,16,17,18,19,20,21,22]. The observed association between P_max_ and critical velocity (r = 0.493) reinforces this interpretation, as improvements in muscular power may enhance neuromuscular recruitment and mechanical efficiency [23]. Moreover, Olstad et al. [24] reported that increases of 5 to 10% in upper-body power may lead to improvements of 2 to 5% in critical velocity among elite swimmers, further supporting the present findings. This relationship is also consistent with results from Sorgente et al. [25], who reported significant associations between load–velocity profiling and middle-distance swimming performance.

In contrast, the strong correlation observed between F_0_ and anaerobic capacity (r = 0.842) highlights the importance of maximal force in sustaining effort beyond critical velocity, a defining feature of anaerobic performance. This aligns with previous research by Zacca et al. [16] and West et al. [26], who reported that swimmers with larger maximal force production demonstrate greater anaerobic capacity values and superior sprint performance (≤100 m). The limited contribution of V_0_ to anaerobic capacity observed in the present study suggests that tolerance to supramaximal effort may rely more on the ability to generate high propulsive forces per stroke rather than on movement velocity. This interpretation is coherent with physiological models positioning anaerobic capacity as an energetic reservoir predominantly reflecting phosphocreatine turnover, anaerobic glycolysis, and maximal neuromuscular output [27]. This divergence in determinants between critical velocity and anaerobic capacity is consistent with the distinction previously described for anaerobic critical speed [28].

Taken together, these findings support the notion that an optimal load–velocity profile in swimming is influenced by race-specific demands [29,30]. Swimmers presenting a greater velocity- and power-oriented profile appear better suited for longer distances via enhanced mechanical efficiency, whereas those with greater maximal force capacity may be advantaged in short-distance events where anaerobic power plays a predominant role [23,31]. This interpretation aligns with current recommendations regarding individualized training and performance-specific periodization in high-level swimming [32].

From a practical standpoint, these findings suggest that interventions aiming to improve critical velocity should prioritize neuromuscular strategies targeting contractile velocity and power, such as explosive strength training and individualized load–V optimization [9,10]. Conversely, enhancing anaerobic capacity may require training oriented toward maximal strength development through high-load protocols, maximal isometric work, or structured progressive loading [11]. Load–velocity profiling therefore offers a relevant framework for guiding dryland training according to the physiological demands of competitive events [24,25].

Certain limitations should be acknowledged. The sample consisted exclusively of trained male swimmers, which limits the generalizability of the findings to female athletes or elite populations. Additionally, biomechanical swimming variables were not assessed, which may have provided further insight into the mechanisms linking the force–velocity profile with critical velocity and anaerobic capacity [4]. Future research should include larger and mixed-gender samples and incorporate biomechanical indicators such as stroke rate, energy cost, and swimming index to better elucidate the observed associations. Moreover, the use of a ballistic bench press velocity profile should be considered, as it would minimize bar deceleration and ensure that the propulsive phase is expressed across the entire range of motion potentially leading to stronger and more accurate correlations with swimming performance.

## 5. Conclusions

This study demonstrated that critical velocity and anaerobic capacity are not solely determined by internal physiological capacities but may be influenced by upper-body maximal strength and velocity. V_0_ and P_max_ appear to be significant contributors to critical velocity, whereas F_0_ was strongly associated with anaerobic capacity. These findings reinforce the importance of an integrated approach combining physiological and neuromuscular analyses to guide and individualize dry-land training for competitive swimming. Furthermore, regular monitoring of the force–velocity profile and maximal bench press power can serve as a useful tool for adjusting the volume, load, and periodization of dry-land training based on the evolution of in-water aerobic and anaerobic capacities.

## Figures and Tables

**Table 1:** Descriptive statistics (mean ± standard deviation) for neuromuscular and physiological parameters.

Variable	
F_0_ (N)	600.16 ± 22.18
V_0_ (m/s)	2.87 ± 0.12
P_max_ (W)	430.16 ± 19.38
100 m (s)	29.35 ± 1.43
200 m (s)	66.30 ± 3.23
400 m (s)	140.88 ± 6.90
Critical velocity (m/s)	2.70 ± 0.13
Anaerobic capacity (m)	21.20 ± 1.17

F_0_: theoretical maximal force; V_0_: theoretical maximal velocity; P_max_: maximal power.

**Table 2:** Correlation and regression analysis of neuromuscular variables with critical velocity and anaerobic capacity.

Variable	Critical Velocity			Anaerobic Capacity		
	r	F	*p*-Value	R	F	*p*-Value
F_0_	0.152	0.664	0.422	0.842	68.001	<0.001
V_0_	0.697	26.446	<0.001	0.291	2.582	0.119
P_max_	0.493	8.969	0.006	0.428	6.283	0.018

F_0_: theoretical maximal force; V_0_: theoretical maximal velocity; P_max_: maximal power.

## Data Availability

The original contributions presented in the study are included in the article/Supplementary Material, further inquiries can be directed to the corresponding authors.

## Supplementary Materials

The following supporting information can be downloaded at: https://www.mdpi.com/article/10.3390/sports14010011/s1, Table S1: Descriptive statistics for neuromuscular and physiological parameters of the swimmers (n = 30).
