# Supplementary material for: Relationships Between Dry-Land Load—Velocity Parameters and In-Water Bioenergetic Performance in Competitive Swimmers
Source: Sports (Basel). 2026 Jan 3;14(1):11. doi: 10.3390/sports14010011 (PMC12846203; doi:10.3390/sports14010011)
Supplement: Supplementary file 1 [file sports-14-00011-s001.zip › sports-4028557-supplementary.pdf]

Table S1. Descriptive statistics for neuromuscular and physiological parameters of the swimmers (n=30).

Swimmer	F_0 (N)	V_0 (m/s)	P_{max} (W)	100 m (s)	200 m (s)	400 m (s)	Critical velocity (m/s)	Anaerobic capacity (m)
1	590	2.848	419.83	30.05	67.99	143.86	2.636	20.79
2	588	3.142	461.57	27.16	61.45	130.01	2.917	20.77
3	580	2.918	422.61	29.49	66.76	141.31	2.683	20.88
4	614	2.793	428.91	30.68	69.65	147.6	2.566	21.28
5	575	2.72	391.4	28.07	63.38	134	2.832	20.5
6	600.8	2.85	428.19	30.45	68.62	144.96	2.62	20.23
7	610	2.945	449.26	28.64	65.11	138.05	2.742	21.47
8	618.8	2.685	415.02	31.3	70.82	149.87	2.53	20.81
9	575	2.761	396.69	30.96	69.75	147.33	2.578	20.19
10	614.8	2.944	452.37	29.09	65.86	139.39	2.72	20.88
11	580	3.009	436.31	28.39	64	135.23	2.808	20.29
12	575	2.941	422.69	28.67	64.64	136.58	2.78	20.29
13	580	2.906	421.37	29.35	66.34	140.34	2.703	20.67
14	570.6	2.884	411.57	29.56	66.26	139.66	2.725	19.46
15	569	2.743	390.63	32.63	73.14	154.18	2.468	19.47
16	594.9	2.834	421.59	29.94	66.94	143.87	2.628	21.86
17	586.8	2.865	420.27	30.03	67.73	143.78	2.636	20.95
18	650	2.71	440.38	28	62.95	136.32	2.763	23.31
19	620	2.74	425.7	29.14	64.7	140.13	2.695	22.28
20	605	2.708	409.87	33.14	75.03	161.24	2.339	22.9
21	631.4	2.76	435.77	28.12	64.78	137.86	2.734	23.07
22	630	2.77	436.28	29.16	66.88	141.9	2.662	22.31
23	632	2.839	449.11	29.04	66.94	141.8	2.662	22.51
24	600	2.993	449	27.8	62.72	133.9	2.825	21.74
25	610	3.044	464.66	27.79	62.77	133.92	2.824	21.75
26	607	2.98	452.27	28.04	63.36	135.95	2.777	22.53
27	610	2.819	430.1	30.53	68.73	146.66	2.581	21.49
28	611.8	2.883	440.68	29.09	65.6	139.84	2.707	21.5
29	615	2.96	455.4	28.68	64.82	138.33	2.734	21.84
30	560	3.037	425.18	27.51	61.32	128.38	2.975	18.03

F_0: theoretical maximal force; V_0: theoretical maximal velocity; P_{max}: maximal power
